# Self-reported questionnaires assessing body perception disturbances in adults with chronic non-cancer pain: a scoping review

**DOI:** 10.3389/fpain.2025.1497328

**Published:** 2025-03-06

**Authors:** Marion Dagenais, Charlotte Proulx, Tania Augière, Jean-Sébastien Roy, Catherine Mercier

**Affiliations:** ^1^ Center for Interdisciplinary Research in Rehabilitation and Social Integration (Cirris), CIUSSS de la Capitale-Nationale, Quebec, QC, Canada; ^2^ School of Rehabilitation Sciences, Laval University, Quebec, QC, Canada

**Keywords:** chronic pain, body perception, disturbances, patient-reported questionnaire, adults

## Abstract

**Introduction:**

Body perception disturbances (BPD) are well documented in certain chronic pain populations [e.g., complex regional pain syndrome (CRPS)], while being far less studied in chronic pain as a general condition. The aims of this scoping review are to identify the self-reported questionnaires used to assess BPD in individuals with chronic non-cancer pain and to refine the definition of the BPD construct as used in these questionnaires.

**Methods:**

A search strategy focusing on the concepts of “chronic pain”, “body perception” and “questionnaire” was used across four databases. Each record was screened for eligibility by two independent reviewers, and data extraction was performed by one reviewer and validated by a second reviewer.

**Results:**

Eighty-seven studies were included, comprising 18 different questionnaires—either directly related to BPD or containing relevant items. The three most commonly used questionnaires were the Bath Body Perception Disturbance Scale, the Fremantle Back Awareness Questionnaire, and the Neurobehavioral Questionnaire. Appraisal of the construct derived from the questionnaire items identified five main facets: size, shape, cognitive neglect-like symptoms, proprioceptive awareness, and agency, along with 11 other less frequently addressed facets. The most represented clinical populations were CRPS (40 studies) and chronic low-back pain (20 studies).

**Discussion:**

A variety of self-reported questionnaires are available to assess BPD, but most are diagnosis- or body-region specific. To better assess BPD in individuals with chronic non-cancer pain, a consensus on the general definition and the key facets of the construct is needed.

## 1 Introduction

Body perception is defined as the way one consciously perceives one's own body, which relies on ongoing sensory input and is thought to be a fluid concept influenced by memories, beliefs, and psychological factors (1). Body perception disturbances (BPD) have been reported in several chronic pain populations (2–5). For example, people with complex regional pain syndrome (CRPS) often report distortions in the perception of their affected limb compared to its actual characteristics (e.g., in terms of size, temperature, pressure) (2). They also have difficulty determining how their limb is positioned (3). Some people feel a sense of foreignness toward their painful limb, while others distrust it (4, 5). Similar disturbances have been described in other chronic pain populations such as phantom limb pain (PLP) (6), chronic low-back pain (CLBP) (7, 8), and chronic knee pain (9). However, evidence regarding the presence or absence of BPD is scarce for many chronic pain populations.

One way to assess the presence of BPD is through self-reported questionnaires. However, most available questionnaires are diagnosis-specific, having been developed for specific pain syndromes. For instance, the Fremantle Back Awareness Questionnaire (FreBAQ) was developed for people with CLBP (10), and the Bath Body Perception Disturbance Scale (BBPDS) was developed for people with CRPS (11). Although some self-reported questionnaires have been adapted for other chronic pain populations [e.g., FreSHAQ for shoulder pain (12), FreKAQ for knee pain (13)], their use remains limited to the specific populations for which they were developed due to the wording of the items. This also makes them unsuitable for pain syndromes affecting other parts of the body (e.g., migraine), pain affecting multiple body areas (e.g., fibromyalgia), or for clinical and research settings involving diverse pain populations. Moreover, the lack of a generic measure of BPD precludes comparisons across various populations, especially when the definition of the construct varies from one questionnaire to another. Thus, clarification of the definition of BPD is essential for its effective assessment in diverse populations. This is especially relevant given that chronic pain is now recognized as a disease in its own right (14, 15), and recent literature shows that pain is associated with BPD in different pain syndromes (16).

Previous work on this topic includes a systematic review that identified available tools for assessing BPD in CRPS (17) and a systematic scoping review that identified available tools for assessing explicit and implicit own's body and space perception in painful musculoskeletal disorders and rheumatic diseases (18). However, both reviews cover only a subset of chronic pain conditions within their target populations. Furthermore, the review by Viceconti et al. addresses constructs (somatoperception, body ownership, space perception) that only partially overlap with body perception, leaving a gap in the literature regarding available self-reported questionnaires to assess BPD in chronic pain as a generic condition, rather than diagnosis- or body part-specific.

To address these gaps, the aims of this scoping review are to identify the self-reported questionnaires used to assess BPD in chronic non-cancer pain populations and to refine the definition of the BPD construct.

## 2 Methods

This scoping review was conducted in accordance with the Joanna Briggs Institute methodology for scoping reviews (19).

### 2.1 Search strategy and selection criteria

A search strategy was elaborated with the assistance of an academic health librarian, using keywords from the Title, Abstract, Keywords and index terms of relevant articles. A comprehensive search was then conducted in MEDLINE, CINAHL, PsychInfo and Embase (see Supplementary Appendix S1 for a sample search of MEDLINE). The search strategy comprised three key concepts: (1) Chronic pain; (2) Body Perception; (3) Questionnaire.

An initial search of the databases was performed on January 16th, 2023 and reruns were performed on February 22nd, 2024, and January 16th, 2025, with no date limits. All references were uploaded and deduped in Endnote X20 (Clarivate Analytics, Philadelphia, USA). References were then imported into Covidence (Covidence Systematic Review Software, 2021), an online software designed to facilitate the conduct of systematic and scoping reviews. The screening for eligibility was performed by two independent reviewers using the following inclusion criteria: (1) participants had to be adults (≥18 years old) with chronic non-cancer pain (i.e., pain ≥3 months); (2) at least one self-reported questionnaire was used to assess BPD; (3) body perception was broadly defined as the way one consciously perceives their own body (1) (e.g., the perceived characteristics of the painful body part(s), such as shape, size and temperature, one's perceived ability to locate and move one's body parts in a controlled manner, as well as the feelings of disownership and foreignness about these body parts); (4) original peer-reviewed studies published in French or English. For studies conducted in a heterogeneous sample, at least 50% of the participants had to meet criterion #1 for the paper to be included. Studies that only used approaches such as psychophysical assessments (e.g., quantitative sensory testing), but no questionnaire (criterion #2), were excluded. Finally, according to criterion #3, self-reported questionnaires defining body perception in terms of body image satisfaction or interoceptive awareness were excluded.

Pilot testing was conducted on 20 references for the Title and Abstract screening, and 10 references for the Full text screening. In case of disagreement between the two reviewers, a third party (CM) was involved to make the final decision. A manual search (e.g., screening of the reference lists of all included studies) was also performed to identify additional eligible studies.

### 2.2 Data extraction

Data extraction of included studies was performed by a first reviewer and revised by a second reviewer. The following information was extracted from each included study: Authors, Year, Country in which the study was conducted, Aim(s), Study type (e.g., cross-sectional study, questionnaire development and psychometric testing), Population (sample size, age, sex, chronic pain diagnosis/es), Questionnaire(s) used, Questionnaire items, Other relevant information (e.g., Original questionnaire development study).

## 3 Results

**Figure 1:**
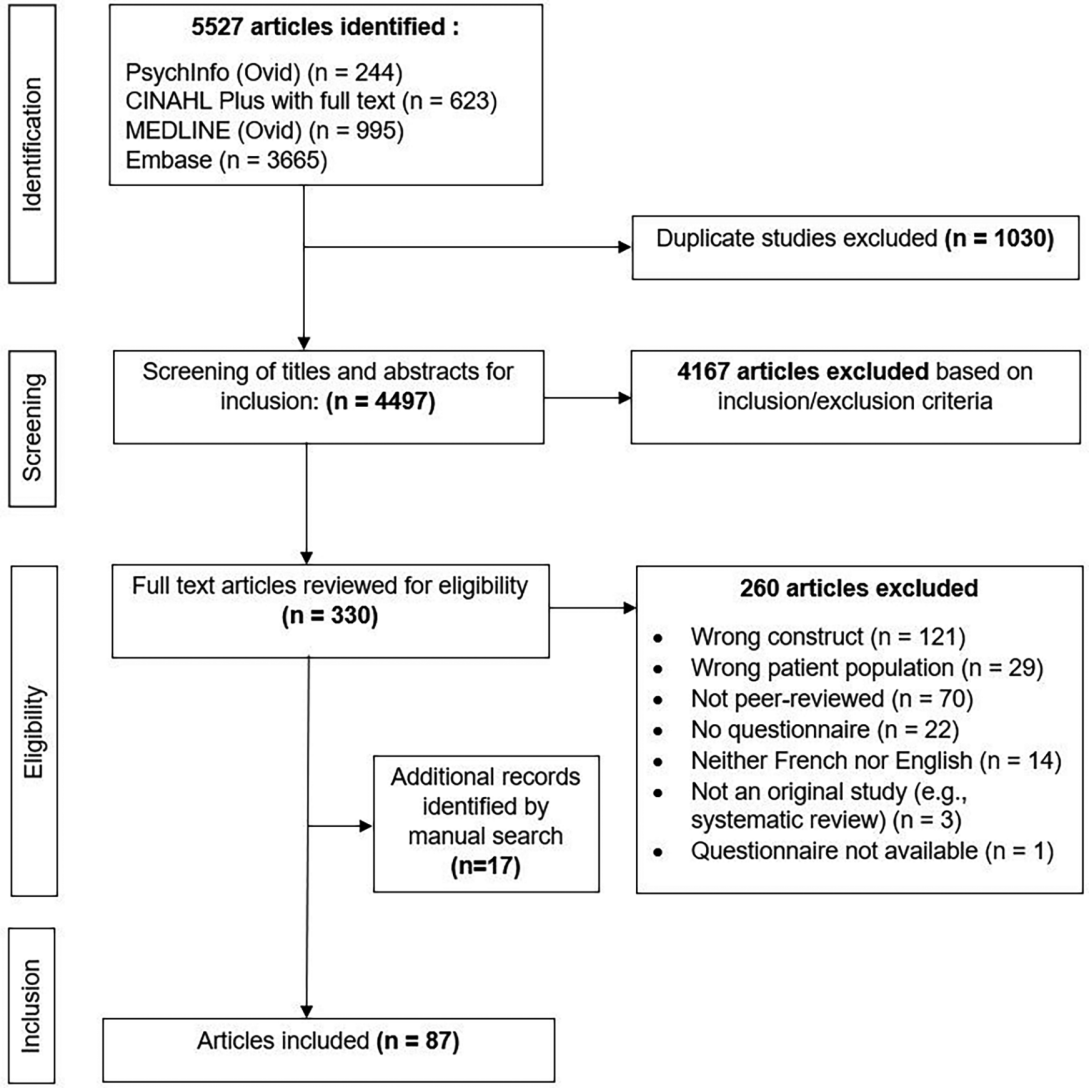
PRISMA flowchart detailing the screening and inclusion process.

The search yielded 5,527 studies. Duplicates were identified and removed using Covidence, leaving 4,497 studies to be screened based on titles and abstracts. Of these, 4,167 studies were excluded, leaving 330 full-text studies to be screened for eligibility. Of these, 70 studies were included. Seventeen additional records were included based on manual search. Thus, a total of 87 studies were included in this review (see Figure 1 for PRISMA flowchart). The included studies were either original studies (*n* = 59) or questionnaire development and/or psychometric validation studies (*n* = 28) (See Supplementary Table S1 for the detailed extraction table of included articles).

### 3.1 Self-reported questionnaires assessing body perception disturbances in chronic pain

Among the 87 studies included in this review, a total of 18 different self-reported questionnaires assessing BPD were identified. These questionnaires were categorized as follows: 3.1.1 standardized questionnaires specifically addressing BPD (*n* = 9); 3.1.2 standardized questionnaires with some items relevant to the construct (*n* = 1); 3.1.3 non-standardized questionnaires specifically addressing BPD (*n* = 5); and 3.1.4 non-standardized questionnaires with some items relevant to the construct (*n* = 3). Questionnaires were considered standardized if they had undergone some level of psychometric testing (e.g., content validity, construct validity, internal consistency), whereas questionnaires that had not been tested (e.g., questionnaires or single questions developed for the purpose of a specific study) were categorized as non-standardized. Tables 1, 2 show the classification of questionnaires and report all the chronic pain populations with which questionnaires were used in the included studies.

**Table 1:** Standardized self-reported questionnaires and chronic pain populations.

Questionnaires	FreBAQ	FreNAQ	FreSHAQ	FreKAQ	FrePAQ	FreBAQ-FM	FreBAQ-general	BBPDS	Neuro behav. Q.	CDS
Chronic pain populations										
Low-back pain	(10,20–28,30–39)								(82)	
Lumbo-pelvic pain	(29)									
Perineal pain					(48)					
Neck Pain		(40–42)								
Shoulder pain			[40,41]							
OA – Knee				(9,13,44–47)					(89)	
OA – Hand									(91,92)	
OA – unspecified								(58)	(58,83,90)	
CRPS								(52–67,70–76,79,80)	(4,5,55,58,60,61,63,77,78,81–88,90)	
Polyneuropathy								(68)	(90)	
Chronic bursitis									(90)	
Enthesopathy									(90)	
PNL									(90)	
Carpal tunnel syndrome								(58)	(58,83)	
Limb pain – unspecified								(58,59)	(58,83–87,90)	
Fracture									(82)	
Migraine									(84)	
Amputation								(69)	(88)	
Chronic pain – unspecified							(51)			
Fibromyalgia						(49,50)			(82)	
Rhumatoid arthritis								(58)	(58,82,83)	
Spinal cord injury										(93)

Chronic pain populations and standardized self-reported questionnaires used in the included studies. The dark blue section includes questionnaires pertaining to body perception disturbances, while the light blue section includes questionnaires comprising some items relevant to the construct. Questionnaires: FreBAQ, Fremantle Back Awareness Questionnaire; FreNAQ, Fremantle Neck Awareness Questionnaire; FreSHAQ, Fremantle Shoulder Awareness Questionnaire; FreKAQ, Fremantle Knee Awareness Questionnaire; FrePAQ, Fremantle Perineal Awareness Questionnaire; FreBAQ-FM; Fremantle Back Awareness Questionnaire-Adaptation for Fibromyalgia; FreBAQ-general, Fremantle Body Awareness-General Questionnaire; BBPDS, Bath CRPS Body Perception Disturbance Scale; Neurobehav. Q., Neurobehavioral Questionnaire; CDS, Cambridge Depersonalization Scale. Chronic pain populations: OA, Osteo-arthritis; CRPS, complex regional pain syndrome; PNL, peripheral nerve lesion. Darker cells indicate pain populations that were the main clinical groups of included studies, while lighter cells indicate pain populations that were included as pain control groups. Included records: Numbers in parentheses correspond to reference numbers for each record—see References section for full citations.

**Table 2:** Non-standardized self-reported questionnaires and chronic pain populations.

Questionnaires	Q. on Body Feelings	MES	Perceived Hand Size	Limb Position Awareness	Feeling of Foreignness	CIBS	Assessment of Neuropathic Pain in FM Patients	Q. on the Phantom Limb
Chronic pain populations								
CRPS	(65)			(3)	(98)			
Oro-facial pain		(96)						
Amputation						(6)		(100)
Fibromyalgia							(99)	
Post-stroke pain			(97)					

Chronic pain populations and non-standardized self-reported questionnaires used in the included studies. The dark orange section includes questionnaires pertaining to body perception disturbances, while the light orange section includes questionnaires comprising some items relevant to the construct. Questionnaires: MES, Magnitude Estimation Scale; CIBS, Changes in body sensation following limb loss Questionnaire; Q. on the Phantom Limb, Questionnaire on the Phantom Limb. Chronic pain populations: CRPS, complex regional pain syndrome. Included records: Numbers in parentheses correspond to reference numbers for each record—see References section for full citations.

#### 3.1.1 Standardized questionnaires pertaining to body perception disturbances

Standardized questionnaires consisting of whole scales developed specifically for the construct include the Fremantle Back Awareness Questionnaire [FreBAQ, *n* = 21 studies (10, 20–39)] and its adaptations for the neck [FreNAQ, *n* = 3 studies (40–42)], shoulder [FreSHAQ, *n* = 2 studies (12, 43)], knee [FreKAQ, *n* = 6 studies (9, 13, 44–47)], perineal region [FrePAQ, *n* = 1 (48)], fibromyalgia [FreBAQ-FM, *n* = 2 (49, 50)], and the region-generic version [FreBAQ-general, *n* = 1 study (51)], as well as the Bath Body Perception Disturbance Scale [BBPDS, *n* = 29 studies (52–80)], and the Neurobehavioral Questionnaire [*n* = 19 studies (4, 5, 55, 58, 60, 61, 63, 81–92)].

The FreBAQ consists of nine items [six items for the FreBAQ-general (51)] and was developed to assess BPD in CLBP. The authors define body perception as “the feelings we have of our own body”. Disturbances include signs of cognitive and motor neglect, a loss of proprioceptive awareness, and a distorted perception of the back in terms of size and delineation (10). Each item is scored on a 5-point Likert-type scale and the total score corresponds to the sum of all the items (maximum score: 36 for the original FreBAQ and the region-specific adaptations, and 24 for the region-generic adaptation), with higher scores reflecting greater disturbances.

The BBPDS consists of six items and a body drawing to assess BPD in CRPS (11). Body perception is defined as “the subjective perception of the affected body part” and disturbances include a feeling of foreignness toward the painful body part, an altered awareness of limb position, strong negative emotions, and an altered perception of the body part in terms of shape, size, weight, and temperature (11, 52). The original scale also comprises an item that assesses attention to the affected limb—disturbances manifesting as either hypervigilance or neglect toward the affected limb. Note that a revised version of the scale excluding the item on attention has been proposed by Ten Brink and collaborators. The authors based their decision on the corrected item-total correlation for this item, which was found to be insufficient (60). Items 1 to 4 and 6b are scored on a 0–10 numerical rating scale, while items 5 and 6a are dichotomous. Finally, the body drawing is scored on a 3-point scale (0 = no distortion; 1 = distortion; 2 = severe distortion). The final score corresponds to the sum of all items plus the body drawing (maximum score: 57), with higher scores reflecting greater disturbances.

The Neurobehavioral Questionnaire, or Neglect-Like Symptoms Questionnaire, is a 5-item questionnaire developed by Galer and Jensen to assess neglect-like symptoms (NLS) in CRPS (4). According to the authors, NLS include cognitive neglect (i.e., perceiving the affected limb as foreign and not part of the body), motor neglect (i.e., having to mentally and visually focus to move the limb), and the presence of involuntary movements. The original scale consists of dichotomous Yes/No items. The total score corresponds to the sum of all items, with higher scores reflecting greater disturbances.

#### 3.1.2 Standardized questionnaires comprising relevant items

This category comprises one questionnaire (*n* = 1 study (93)). The Cambridge Depersonalization Scale (CDS), a standardized questionnaire developed by Sierra and Berrios to assess depersonalization, includes some items related to BPD (relevant items are listed in Supplementary Table S1) (94). Each of the 29 items of the CDS requires a dual-scoring: the “Frequency” (5-point adjectival scale) and “Duration” (6-point adjectival scale) of each phenomenon. The CDS defines depersonalization as “an alteration in the perception or experience of the self so that one feels detached from, and as if one is an outside observer of, one's mental processus or body” and “an alteration in the perception or experience of the external world so that it seems strange or unreal”. Moreover, the scale accounts for phenomena such as “heightened self-observation”, “changes in body experience”, and “changes in the feeling of agency”. Scores are added separately for “Frequency” and “Duration”, then both totals are combined to obtain a final score. Higher scores reflect greater levels of depersonalization.

#### 3.1.3 Non-standardized questionnaires assessing body perception disturbances

This category comprises one questionnaire [Questionnaire on Body Feelings, *n* = 1 study (65)] and four single questions, either open-ended or dichotomous.

The Questionnaire on Body Feelings was developed by Tajadura-Jiménez et al. to assess participants' perceived body behavior. It was originally developed for a study with pain-free individuals (95) and consists of eight items scored on 7-point scales. The first four items use adjectival scales to assess the perceived speed, weight, strength, and extension of the body, while the remaining four items use Likert scales to assess the feelings of agency, vividness, surprise and feet localization. There is no total score for this questionnaire.

Two studies used single questions to assess the perceived size of a body part. Dagsdóttir et al. assessed the perceived distortion of the face by asking participants with oro-facial pain whether they perceived their face to be either swollen or reduced in size (96). In addition, participants rated their perceived distortion on a Magnitude Estimation Scale (MES) ranging from −100% to +100% (−100% = half the size, 0 = no change, + 100% = double the size). Haslam et al. assessed the perceived change in hand size in individuals with chronic post-stroke pain using a yes/no question (“Since your stroke, does it feel like your hand is now a different size?”) (97). If participants answered “yes”, a follow-up question asked whether their hand felt larger or smaller.

One study used a single open-ended question to assess the awareness of limb position in participants with CRPS (“On a daily basis, how aware are you of the position of your limbs?”) (3).

One study used a single open-ended question to assess the feeling of foreignness toward the affected hand in participants with upper-limb CRPS (98). Participants were asked how they felt about their hand. If participants were unsure of the meaning of the question, a series of close-ended questions were asked to determine whether participants perceived their hand as “ill”, “foreign”, “clumsy”, “unsuitable” or “strange”.

#### 3.1.4 Non-standardized questionnaires comprising relevant items

This category comprises three questionnaires: the “Changes in body sensation following limb loss” Questionnaire (CIBS-questionnaire, *n* = 1 study (6)), the “Assessment of neuropathic pain in fibromyalgic patients” (*n* = 1 study (99)) and the “Questionnaire on the Phantom Limb” (*n* = 1 study (100)).

The CIBS-questionnaire was developed by Giummarra et al. to explore different aspects of the phantom limb (e.g., perceived size, shape and posture of the phantom limb, ability to move the phantom limb, PLP) following amputation (6). It consists of 84 items, some of which relate to aspects of “body perception” as defined by Lotze and Moseley (relevant items are identified in Supplementary Table S1). The authors defined the purpose of the questionnaire as “an exploration of the perception of somatic and other qualities in the phantom limb following amputation”. There is no total score for this questionnaire.

The “Assessment of neuropathic pain in fibromyalgic patients” is an online survey developed by Viceconti et al. to explore neuropathic pain symptoms in participants with fibromyalgia. It consists of 37 items, including three items assessing body perception disturbances (99). Participants were provided with a list of 19 body parts and had to select all the body parts in which they experienced pain or stiffness. Then, for each symptomatic area, participants were asked whether they experienced illusory perceptions of swelling, shrinkage, asymmetry, a feeling of constriction or heaviness across that area (first item), how long they had experienced these disturbances (second item), and whether they had mentioned this phenomenon to their health care professionals (third item).

The “Questionnaire on the Phantom Limb” was developed by Kooijman et al. to report on phantom sensations (three items), phantom pain (five items), and stump pain (six items) (100). Among the phantom sensations items, one item lists descriptive words to express the quality of the phantom sensations (e.g., movement, abnormal shape, abnormal, position), which is related to BPD and was therefore included in the present review.

### 3.2 Language versions

Several language versions of the questionnaires were found (see Tables 3, 4). All 18 questionnaires were available in English—the questionnaires were either originally developed in English, or an English version can be found in the included records (e.g., while the FreNAQ is available in Turkish (41) and Japanese (40), the authors also included an English version of the questionnaire in the published paper). For one of the questionnaires included in this review (CDS), the French version of the questionnaire was used in the included record (93). However, the questionnaire was originally developed in English (94) and is therefore available in this language. Some linguistic adaptations were performed through formal forward-backward translation processes, while others were informally translated by research teams for the purposes of their study (these data are available in Supplementary Table S1).

**Table 3:** Standardized self-reported questionnaires and available language versions.

Questionnaires	FreBAQ	FreNAQ	FreSHAQ	FreKAQ	FrePAQ	FreBAQ-FM	FreBAQ-general	BBPDS	Neuro behav. Q.	CDS
Languages										
English	(10,29,34,35)	(40–42)	(12,43)	(13,44–46)	(48)	(49)	(51)	(52,57–66,69,72,74–79)	(58,60,61,63,82,86,91,92)	(94)
Turkish	(20)	(41)								
Dutch	(21,30)									
German	(24,31,32)							(55,67,68)	(5,55,83–85,87,88,90)	
Japanese	(26,33,36,37)	(40)	(43)	(9,13,46,47)				(56,73)	(81,89)	
Persian	(22)			43						
Italian	(39)			42						
French							(51)	(53,54,80)		(93)
Korean								(70)		
Ukrainian								(71)		
Chinese	(23)									
Indian	(25)									
Greek		(42)	(12)							
Spanish	(27,28,38)					(49,50)				
Arabic	(38)									

Available language versions of standardized self-reported questionnaires. The dark blue section includes questionnaires pertaining to body perception disturbances, while the light blue section includes questionnaires comprising some items relevant to the construct. Questionnaires: FreBAQ, Fremantle Back Awareness Questionnaire; FreNAQ, Fremantle Neck Awareness Questionnaire; FreSHAQ, Fremantle Shoulder Awareness Questionnaire; FreKAQ, Fremantle Knee Awareness Questionnaire; FrePAQ, Fremantle Perineal Awareness Questionnaire; FreBAQ-FM, Fremantle Back Awareness Questionnaire-Adaptation for Fibromyalgia; FreBAQ-general, Fremantle Body Awareness-General Questionnaire; BBPDS, Bath CRPS Body Perception Disturbance Scale; Neurobehav. Q., Neurobehavioral Questionnaire; CDS, Cambridge Depersonalization Scale. Darker cells indicate available language versions of the questionnaires, while lighter cells indicate that an informal English version is available in the included records. Included records: Numbers in parentheses correspond to reference numbers for each record—see References section for full citations.

**Table 4:** Non-standardized self-reported questionnaires and available language versions.

Questionnaires	Questionnaire on body feelings	MES	Perceived hand size	Limb position awareness	Feeling of foreignness	CIBS	Assessment of neuropathic pain in FM patients	Q. on the phantom limb
Languages								
English	(65)	(96)	(97)	(3)	(98)	(6)	(99)	(100)
Dutch								(100)
German					(98)			
Italian							(99)	
Danish		(96)						

Available language versions of non-standardized self-reported questionnaires. The dark orange section includes questionnaires pertaining to body perception disturbances, while the light orange section includes questionnaires comprising some items relevant to the construct. Questionnaires: MES, Magnitude Estimation Scale; CIBS, Changes in body sensation following limb loss Questionnaire; Q. on the Phantom Limb, Questionnaire on the Phantom Limb. Darker cells indicate available language versions of the questionnaires, while lighter cells indicate that an informal English version is available in the included records. Included records: Numbers in parentheses correspond to reference numbers for each record—see References section for full citations.

### 3.3 Chronic pain populations

#### 3.3.1 Clinical groups

Table 1 outlines the clinical populations in which the questionnaires were used in the included studies. The most represented clinical populations were CRPS (*n* = 40), CLBP (*n* = 20), and OA (*n* = 9; hand OA: *n* = 2, knee OA: *n* = 7). Other diagnoses included amputation (*n* = 4), chronic neck pain (*n* = 3), fibromyalgia (*n* = 3), and chronic shoulder pain (*n* = 2). Finally, lumbo-pelvic pain, perineal pain, chronic pain of unspecified origin, polyneuropathy, oro-facial pain, post-stroke pain, and spinal cord injury were each represented in one study.

#### 3.3.2 Pain control groups

Among the studies that recruited participants with CRPS, some also recruited participants with other chronic pain conditions to form a “pain control group”. For example, one study recruited participants with CLBP, fracture, fibromyalgia, and rheumatoid arthritis (82). Another study recruited participants with OA (unspecified site), polyneuropathy, chronic bursitis, enthesopathy, peripheral nerve injury, and “limb pain other than CRPS (unspecified origin)” (90). Two studies recruited pain control participants with OA (unspecified site), carpal tunnel syndrome, rheumatoid arthritis, and “limb pain other than CRPS (unspecified origin)” (58, 83). Finally, five studies included participants with “limb pain other than CRPS”, without further specification.

### 3.4 “body perception disturbances”—appraisal of the construct based on questionnaire items

**Figure 2:**
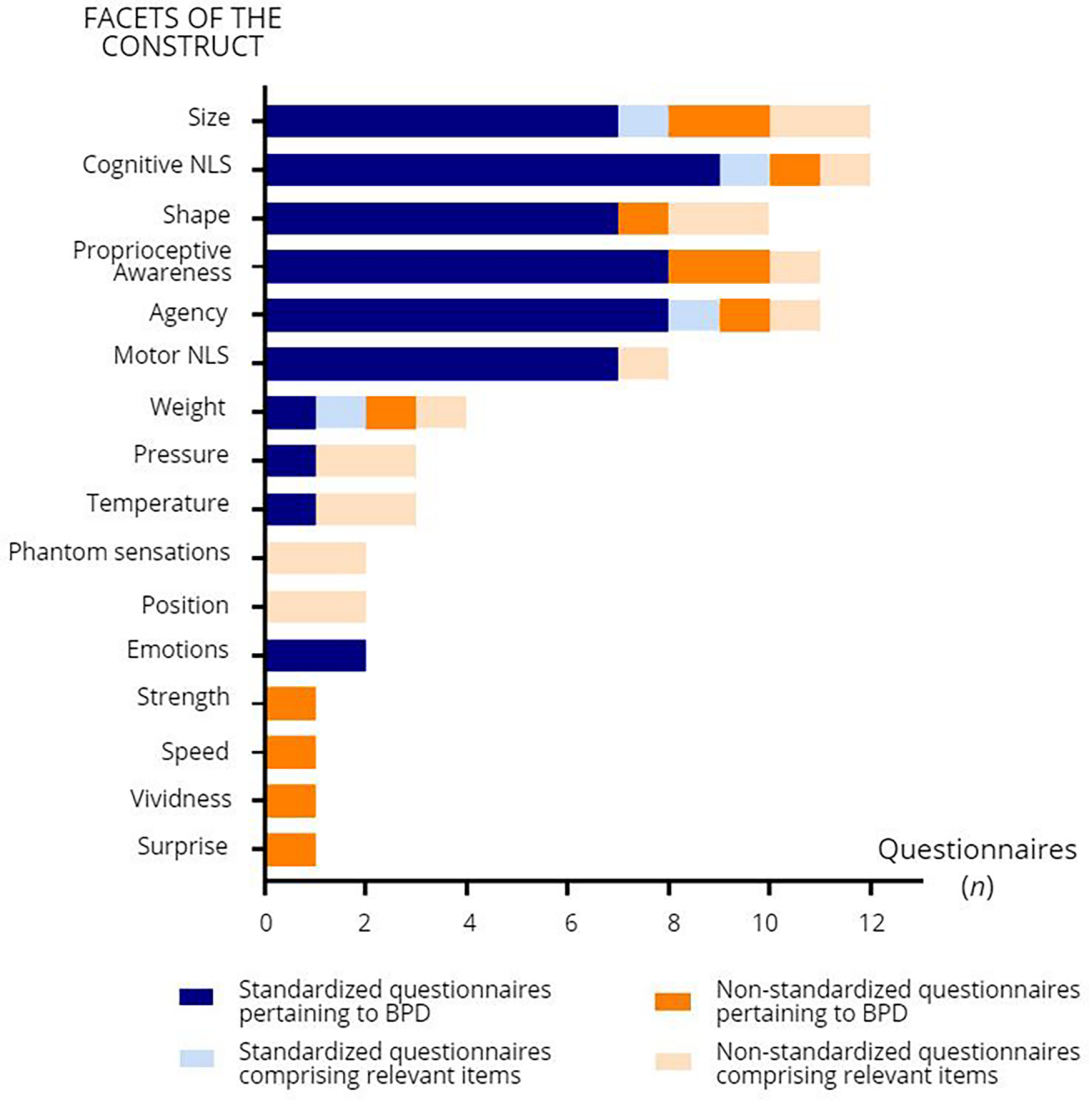
Facets of body perception disturbances (BPD) addressed in the self-reported questionnaires and number of questionnaires (n) comprising items related to these facets. NLS: neglect-like symptoms.

In order to gain a clearer view of how the construct of “body perception disturbances” is defined in chronic pain, the items of each questionnaire were compiled and sorted according to the underlying aspect of the construct that they assessed. Figure 2 shows the different aspects of the construct and how often they were addressed in the questionnaires included in the present review (see Supplementary Table S2 for a detailed compilation of which aspects were addressed in which questionnaire). The most frequently addressed aspects of the construct were the perceived size of the painful body part (*n* = 12 questionnaires, e.g., from the FreBAQ: “My back feels like it is enlarged”, “My back feels like it has shrunk”), cognitive NLS (*n* = 12 questionnaires, e.g., from the Neurobehavioral Questionnaire: “My painful limb feels as though it is *not* part of the rest of my body”), the perceived shape of the painful body part (*n* = 11 questionnaires, e.g., from the FreBAQ: “My back feels lopsided (asymmetrical)”), proprioceptive awareness (*n* = 11 questionnaires, e.g., from the Bath BPDS: “On a scale of 0–10 how aware are you of the physical position of your limb?”), and agency (*n* = 11 questionnaires, e.g., from the CDS: “When I move it doesn't feel as if I were in charge of the movements, so that I feel “automatic” and mechanical as if I were a “robot” ”). Other aspects of the construct included motor NLS (*n* = 8 questionnaires, e.g., from the Neurobehavioral Questionnaire: “I need to focus all my attention on my painful limb to make it move the way I want it to”), and perceived weight (*n* = 4 questionnaires), pressure (*n* = 3 questionnaires), and temperature (*n* = 3 questionnaires) of the painful body part (e.g., from the Bath BPDS: “Is there a difference between how your affected limb looks or is on touch compared to how it feels to you in terms of the following: Size, Temperature, Pressure, Weight”). Emotions toward the painful body part were assessed in two questionnaires (e.g., from the Bath BPDS: “On a scale of 0–10 how strong are the emotional feelings that you have about your limb?”).

Some aspects of the construct were only addressed in certain clinical populations. For example, the perceived position of the phantom limb (*n* = 2 questionnaires, e.g., from the CIBS-questionnaire: “Where does your phantom limb usually sit relative to your other limbs?”), and the presence of phantom sensations of itching, touching, electric or vibration sensations (*n* = 2 questionnaires, e.g., for the CIBS-questionnaire: “Which phantom sensations do you experience?”, response options including “Itching in or on the phantom”, “Something touching the phantom”, and “Electric or vibration sensations”) were explored only in questionnaires specifically designed for amputees. Finally, perceptions of speed (e.g., “I feel slow” to “I feel quick”, 7-point adjectival scale), strength (e.g., “I feel weak” to “I feel strong”, 7-point adjectival scale), vividness (e.g., “It seems the feeling of my body is less vivid than normal”), and surprise (e.g., “The feelings about my body are surprising and unexpected”) were assessed only in the Questionnaire on body feelings, which was used in a study with CRPS participants.

## 4 Discussion

The aims of this scoping review were to identify the self-reported questionnaires used to assess BPD in individuals with chronic non-cancer pain and to refine the definition of the BPD construct as used in these questionnaires. To our knowledge, this is the first literature review that aimed to identify questionnaires assessing BPD in individuals with chronic pain, transcending specific diagnoses. Eighteen questionnaires were identified, with the BBPDS, FreBAQ and Neurobehavioral Questionnaire being the most commonly used. While some pain populations were represented in several studies (e.g., CRPS, CLBP), there was an overall lack of diversity in terms of pain conditions. In addition, this scoping review attempted to circumscribe the main facets of BPD in chronic pain, drawing from identified questionnaires in the literature, in order to find a common terminology and definition for this construct in chronic pain. This process allowed us to identify five main facets at the core of the construct.

### 4.1 Body perception disturbances: beyond a diagnosis

The studies included in this review investigated a variety of pain conditions, with CRPS being the most common, followed by CLBP. This is not surprising given the extensive literature on BPD in CRPS (2, 4, 17, 101) and CLBP (7, 8, 102). Quite expectedly, the three most commonly used questionnaires were developed specifically for these pain conditions. As for PLP, the paucity of included studies on this condition may seem surprising given the abundance of literature in this field (1, 103, 104). However, this could be explained by the decision to focus on questionnaires in the present review. Therefore, studies using outcome measures other than questionnaires (e.g., interviews) were excluded. Notably, there is a glaring scarcity of studies focusing on pain conditions other than the three mentioned above. This could be explained by the fact that most standardized questionnaires are body region- or diagnosis-specific (e.g., FreBAQ and its region-specific adaptations, BBPDS). This creates a vicious circle; there is limited evidence for the presence of BPD in non-specific chronic pain, and there are few available questionnaires to assess this phenomenon in a heterogeneous chronic pain population. Yet, chronic pain has recently been recognized as a disease by the *International Classification of Diseases* (ICD-11) (15). Furthermore, recent evidence suggests that the chronicity of pain is paralleled by the spread of the pain throughout the body (105). Thus, BPD in individuals with chronic pain should be assessed globally, without regard to diagnosis or body region, assuming that BPD also spreads with pain.

Walton et al. assessed BPD in chronic pain without targeting specific conditions by creating a region-generic version of the FreBAQ (51). This version was administered to individuals who self-identified as having chronic pain, with the term “chronic pain” intentionally left vague. This approach allowed for the examination of BPD as an independent construct, not tied to a specific diagnosis or body region. However, Walton et al's study had limitations; chronic pain was investigated in veterans via a survey, and information on pain (onset, intensity, type of pain) and psychosocial factors (e.g., kinesiophobia, catastrophizing, disability) was lacking. This lack of detailed data prevented further analysis of the relationship between BPD and clinical characteristics. In addition, the item wording of the FreBAQ-general is oriented toward the most painful body part, which limits the investigation of BPD to a single body site. This may be of limited use for pain conditions with widespread pain (e.g., fibromyalgia, rheumatoid arthritis). Nevertheless, this questionnaire is a promising first step in the assessment of BPD in chronic pain populations and may allow for the investigation and comparison of the phenomenon across different pain populations in research and clinical settings.

### 4.2 Body perception disturbances: toward a common terminology and definition?

One of the main challenges in assessing BPD in individuals with chronic pain is the lack of a consistent definition for this construct and a standardized terminology to describe it. In fact, authors in the field use different terminologies to refer to the same phenomena, sometimes interchangeably. Notably, some authors speak of “body perception disturbances” (26, 52, 99), “disturbances in body representation” (59), or “impaired self-perception” (10, 46), while others use the terms “maladaptive perceptual awareness” (29), “disturbed body self-awareness” (12, 33) or “distorted body image” (1, 73, 85). Some authors considered NLS to be indicative of the presence of body perceptual disturbances (91) and used the Neurobehavioral Questionnaire (4) or an open-ended question about feelings of foreignness (98) to assess the presence of such symptoms, while others considered NLS to be a subfactor of the construct (along with impaired proprioception and distorted body image) and opted for the FreBAQ (10) or one of its adaptations. In light of this, and to avoid confusion with other constructs (e.g., body awareness, self-perception), we recommend using the terminology “body perception disturbances”.

Despite different terminologies and definitions, certain facets of the construct emerge as essential for a comprehensive understanding of the breadth of BPD in chronic pain. Our findings suggest that the core construct encompasses five main facets: a distorted perception of the size (a) and shape (b) of the painful body part, the presence of cognitive NLS (c), reduced proprioceptive awareness (d), and a disturbed sense of agency (e). Investigation of these facets is paramount to a comprehensive assessment of BPD in individuals with chronic pain. Therefore, studies investigating BPD should select questionnaires that include these facets. Interestingly, however, Walton et al. chose to exclude the two items pertaining to the perceived size of the painful body part from the FreBAQ-general. This decision was based on the results of their exploratory and confirmatory analyses, where the fit for a one-factor model improved after excluding these items (51). One explanation for this could be that these two items [“My (body part) seems larger/smaller than it should be”] were misinterpreted by participants. Indeed, the wording does not necessarily reflect that the painful body part *feels* smaller/larger than it *actually* is, which is at the core of BPD. Thus, content validity could be examined to ensure and, if necessary, improve the comprehensibility of these items (106). This limitation has been acknowledged by the authors.

As for motor NLS, and the perceived weight, pressure, and temperature of the painful body part, they were also considered as relevant facets of the construct in some of the questionnaires. Finally, some aspects of the construct were only investigated in specific pain populations (e.g., the presence of phantom sensations was only assessed in PLP, while perceptions of speed and vividness were only assessed in CRPS), raising the question of whether these facets are relevant in *some* pain conditions, rather than in chronic pain as a global condition.

### 4.3 Methodological considerations

This scoping review has some limitations. One potential limitation is the use of a key concept related to questionnaires (criterion #2: self-reported questionnaires). While studies on questionnaire development and validation were easily identified with our search strategy, original studies using relevant questionnaires did not always include them in the title, abstract, keywords or index terms. As a result, our search strategy may have missed some relevant records. Nevertheless, a manual search identified 17 additional records. Therefore, it is reasonable to assume that most, if not all, relevant records were included in this review. Another potential limitation lies in the construct appraisal. By adopting the definition proposed by Lotze and Moseley (“the way one's body feels to its owner”) to inform the eligibility of records for this review (criterion #3: body perception), one cannot exclude the possibility of selection bias in identifying key facets of the construct. In fact, we chose to exclude questionnaires pertaining to constructs deemed different from “body perception”. In the interest of consistency, we chose to focus our research on questionnaires developed under a common construct, rather than including different constructs that would only add to the existing confusion around the operational definition of “body perception disturbances”. Finally, the psychometric properties of the identified questionnaires were not assessed, as this was beyond the scope of the present review. Therefore, the next steps should include a systematic critical appraisal of the psychometric properties of each questionnaire to enable clinicians and researchers to make informed choices when selecting self-reported questionnaires.

## 5 Conclusion

This scoping review contributes to the field of BPD in chronic pain by identifying available self-reported questionnaires and by attempting to refine the construct definition, while also recommending a unified terminology. As visions of chronic pain evolve, it is paramount to assess how this condition affects body perception and to gain a better understanding of the similarities and differences in the experience of BPD in chronic pain of different origins. The use of common terminology and a generic self-reported questionnaire to assess BPD in chronic pain should allow to portray BPD as a construct independent of specific pain conditions. Further research should include validation studies for the FreBAQ-general in heterogeneous pain populations—including content validity for both the 6-item and the 9-item versions, given that the latter assesses all five main facets of BPD—and descriptive studies in large samples to gain a more complete picture of BPD in chronic pain.

## Data Availability

The original contributions presented in the study are included in the article/Supplementary Material, further inquiries can be directed to the corresponding author.
