# Supplementary material for: Self-reported questionnaires assessing body perception disturbances in adults with chronic non-cancer pain: a scoping review
Source: Front Pain Res (Lausanne). 2025 Mar 6;6:1497328. doi: 10.3389/fpain.2025.1497328 (PMC11922727; doi:10.3389/fpain.2025.1497328)
Supplement: Supplementary file 1 [file Table1.docx]

| 1 | (Bod* adj (image or identity or representation* or schema* or perception* or distortion* or ownership or disownership or awareness)).ti. or (Bod* adj (image or identity or representation* or schema* or perception* or distortion* or ownership or disownership or awareness)).ab. | 17073 |
| --- | --- | --- |
| 2 | (Self adj (concept or awareness)).ti. or (Self adj (concept or awareness)).ab. | 10609 |
| 3 | ("Peripersonal space*" or "Space perception*" or "Extrapersonal space*" or "Personal space*" or "PPS" or "Spatial perception*").ti. or ("Peripersonal space*" or "Space perception*" or "Extrapersonal space*" or "Personal space*" or "PPS" or "Spatial perception*").ab. | 10581 |
| 4 | ("Multisensory integration*" or "Action* representation*").ti. or ("Multisensory integration*" or "Action* representation*").ab. | 2362 |
| 5 | ("Embod*" or "Disembod*" or "Size perception*" or "Somesthetic Perception*" or "anomalous perception*" or "perception anomal*").ti. or ("Embod*" or "Disembod*" or "Size perception*" or "Somesthetic Perception*" or "anomalous perception*" or "perception anomal*").ab. | 16716 |
| 6 | ("Sensory neglect*" or "Spatial neglect*" or "hemisensory neglect*" or "hemispatial neglect*" or "unilateral neglect*" or "neglect-like").ti. or ("Sensory neglect*" or "Spatial neglect*" or "hemisensory neglect*" or "hemispatial neglect*" or "unilateral neglect*" or "neglect-like").ab. | 1351 |
| 7 | ((discriminat* or percept* or somatosensory or "somatic sensation*") adj dis*).ti. or ((discriminat* or percept* or somatosensory or "somatic sensation*") adj dis*).ab. | 4080 |
| 8 | (Body image or Self Concept or Perceptual disorders or Size perception or Space perception or Personal space).sh. | 114608 |
| 9 | 1 or 2 or 3 or 4 or 5 or 6 or 7 or 8 | 158935 |
| 10 | ("Self Report*" or "Survey*" or "Questionnaire*" or "Scale*").ti. or ("Self Report*" or "Survey*" or "Questionnaire*" or "Scale*").ab. | 2456562 |
| 11 | Surveys and Questionnaires.sh. | 580463 |
| 12 | Self Report.sh. | 45392 |
| 13 | 10 or 11 or 12 | 2599935 |
| 14 | ((chronic or widespread or diffuse or persist* or recurr*) adj pain*).ti. or ((chronic or widespread or diffuse or persist* or recurr*) adj pain*).ab. | 59983 |
| 15 | ("back pain*" or "lumbago" or "back ache*" or "backache*" or "vertobrogenic pain*").ti. or ("back pain*" or "lumbago" or "back ache*" or "backache*" or "vertobrogenic pain*").ab. | 64029 |
| 16 | ("neck pain*" or "neck ache*" or "cervicalgia*" or "neckache*" or "cervical pain*").ti. or ("neck pain*" or "neck ache*" or "cervicalgia*" or "neckache*" or "cervical pain*").ab. | 13987 |
| 17 | ("Complex regional pain syndrome*" or "Causalgia" or "CRPS" or "reflex sympathetic dystrophy" or "algodystroph*").ti. or ("Complex regional pain syndrome*" or "Causalgia" or "CRPS" or "reflex sympathetic dystrophy" or "algodystroph*").ab. | 6839 |
| 18 | ("phantom pain*" or "phantom limb*").ti. or ("phantom pain*" or "phantom limb*").ab. | 2322 |
| 19 | ("Fibromyalgia*" or "Fibromyositis" or "Muscular rheumatism*" or "fibrositis*" or "myofascial pain*" or "chronic fatigue" or "trigger point*").ti. or ("Fibromyalgia*" or "Fibromyositis" or "Muscular rheumatism*" or "fibrositis*" or "myofascial pain*" or "chronic fatigue" or "trigger point*").ab. | 25019 |
| 20 | (pain adj (disorder* or syndrome*)).ti. or (pain adj (disorder* or syndrome*)).ab. | 21955 |
| 21 | ("osteoarthr*" or "arthros*" or "degenerative arthriti*" or "arthralgi*" or "polyarthralgia*" or "arthrodyni*").ti. or ("osteoarthr*" or "arthros*" or "degenerative arthriti*" or "arthralgi*" or "polyarthralgia*" or "arthrodyni*").ab. | 150961 |
| 22 | ("Neuralgia*" or "neuropathic pain*" or "neurodynia*" or "nerve pain*" or "musculoskeletal pain*" or "myalgia*" or "muscle pain*" or "soreness*" or "tenderness*").ti. or ("Neuralgia*" or "neuropathic pain*" or "neurodynia*" or "nerve pain*" or "musculoskeletal pain*" or "myalgia*" or "muscle pain*" or "soreness*" or "tenderness*").ab. | 86467 |
| 23 | ("knee pain*" or "hip pain*" or "shoulder pain*" or "lumbopelvic pain*" or "pelvic pain*" or "elbow pain*" or "limb* pain*").ti. or ("knee pain*" or "hip pain*" or "shoulder pain*" or "lumbopelvic pain*" or "pelvic pain*" or "elbow pain*" or "limb* pain*").ab. | 39298 |
| 24 | ("hyperalg*" or "allodyni*" or "hyperesthe*" or "hypesthesia*" or "hypoesthesi*" or "numbness" or "reduced sensation*" or "impaired sensation*" or "paresthes*" or "dysesthes*").ti. or ("hyperalg*" or "allodyni*" or "hyperesthe*" or "hypesthesia*" or "hypoesthesi*" or "numbness" or "reduced sensation*" or "impaired sensation*" or "paresthes*" or "dysesthes*").ab. | 49183 |
| 25 | (Chronic pain or Low back pain or Back pain or Neck pain or Complex regional pain syndromes or causalgia or Reflex sympathetic dystrophy or Phantom limb or Fibromyalgia).sh. | 91091 |
| 26 | (Fatigue syndrome, chronic or Myofascial pain syndromes or Temporomandibular Joint Dysfunction Syndrome or Osteoarthritis or Osteoarthritis, Hip or Osteoarthritis, Knee or Osteoarthritis, Spine or Shoulder pain or Neuralgia or Musculoskeletal pain or Myalgia or Pelvic pain or Somatosensory Disorders or Hyperalgesia or Hyperesthesia or Hypesthesia or Paresthesia).sh. | 153579 |
| 27 | 14 or 15 or 16 or 17 or 18 or 19 or 20 or 21 or 22 or 23 or 24 or 25 or 26 | 501412 |
| 28 | 9 and 13 and 27 | 904 |
