# Supplementary material for: Self-reported questionnaires assessing body perception disturbances in adults with chronic non-cancer pain: a scoping review
Source: Front Pain Res (Lausanne). 2025 Mar 6;6:1497328. doi: 10.3389/fpain.2025.1497328 (PMC11922727; doi:10.3389/fpain.2025.1497328)
Supplement: Supplementary file 3 [file Table3.docx]

Table S2: Facets of the construct assessed in the self-reported questionnaires


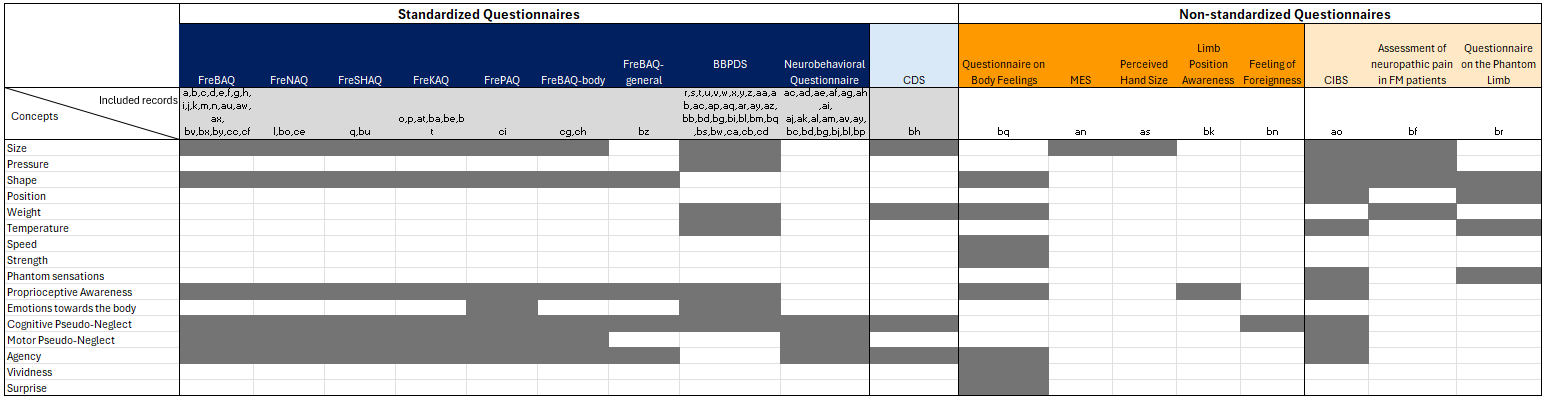


Facets of body perception disturbances addressed in the different questionnaires. The dark blue section includes questionnaires pertaining to body perception disturbances, while the light blue section includes questionnaires comprising some items relevant to the construct. The dark orange section includes questionnaires pertaining to body perception disturbances, while the light orange section includes questionnaires comprising some items relevant to the construct.

**PROMs**: **FreBAQ**: Fremantle Back Awareness Questionnaire; **FreNAQ**: Fremantle Neck Awareness Questionnaire; **FreSHAQ**: Fremantle Shoulder Awareness Questionnaire; **FreKAQ**: Fremantle Knee Awareness Questionnaire; **FrePAQ**: Fremantle Perineal Awareness Questionnaire; **FreBAQ-FM**: Fremantle Back Awareness Questionnaire – Adaptation for Fibromyalgia; **FreBAQ-general**: Fremantle Body Awareness-General Questionnaire; **BBPDS**: Bath CRPS Body Perception Disturbance Scale; **MES**: Magnitude Estimation Scale; **CDS**: Cambridge Depersonalization Scale; **CIBS**: Changes in body sensation following limb loss Questionnaire.

**Included records**:

^a^ (Wand et al., 2014); ^b^ (Erol et al., 2019); ^c^(Janssens et al., 2017); ^d^(Schafer et al., 2021); ^e^(Beales et al., 2016); ^f^(Ehrenbrusthoff et al., 2018); ^g^(Nishigami et al., 2018); ^h^(Rabey et al., 2016); ^i^(Wand et al., 2016); ^j^(Shigetoh et al., 2020); ^k^(Yamashita et al., 2019); ^l^(Yamashita et al., 2021); ^m^(Goossens et al., 2019); ^n^(Mahmoudzadeh et al., 2020); ^o^(Nishigami et al., 2017); ^p^(Monticone et al., 2021); ^q^(Nishigami et al., 2021a); ^r^(Lewis et al., 2012); ^s^(Brun et al., 2018); ^t^(Bultitude et al., 2017); ^u^(Hwang et al., 2014); ^v^(Kotiuk et al., 2019); ^w^(Lewis et al., 2019); ^x^(Osumi et al., 2015); ^y^(Ryan et al., 2017); ^z^(Vittersø et al., 2020); ^aa^(Lewis et al., 2021); ^ab^(Echalier et al., 2020); ^ac^(Schulte-Goecking et al., 2020); ^ad^(Galer and Jensen 1999); ^ae^(Frettlöh et al., 2006); ^af^(Hayashi et al., 2016); ^ag^(Hirakawa et al., 2014); ^ah^(Kuttikat et al., 2017); ^ai^(Magni et al., 2018); ^aj^(Kolb et al., 2012); ^ak^(Michal et al., 2017); ^al^(Reinersmann et al., 2013); ^am^(Wittayer et al., 2018); ^an^(Dagsdottir et al., 2016); ^ao^(Giummarra et al., 2010); ^ap^(Mibu et al., 2021); ^aq^(Beisheim-Ryan et al., 2022); ^ar^(Halicka et al., 2021a); ^as^(Haslam et al., 2022); ^at^(Hedayati et al., 2022); ^au^(Hu et al., 2022); ^av^(Magni et al., 2021); ^aw^(Meier et al., 2021); ^ax^(Rao et al., 2021); ^ay^(Reinersmann et al., 2021); ^az^(Steenkeen et al., 2023); ^ba^(Tanaka et al., 2021); ^bb^(Ten Brink et al., 2021a); ^bc^(Ten Brink et al., 2021b); ^bd^(Ten Brink et al., 2021c); ^be^(Toda et al., 2021); ^bf^(Viceconti et al., 2022); ^bg^(Vittersø et al., 2021a); ^bh^(Pozeg et al., 2017); ^bi^(Halicka et al., 2020); ^bj^(Reinersmann et al., 2012); ^bk^(Lewis et al., 2010); ^bl^(Vittersø et al., 2021b); ^bm^(Bultitude et al., 2021); ^bn^(Förderreuther et al., 2004); ^bo^(Onan et al., 2019); ^bp^(Reinersmann et al., 2010); ^bq^(Tajadura-Jimenez et al., 2017); ^br^(Kooijman et al., 2000); ^bs^(Halicka et al., 2021b); ^bt^(Nishigami et al., 2021b); ^bu^(Koumantakis et al., 2023); ^bv^(Kurashima et al., 2023); ^bw^(De Schoenmacker et al., 2023); ^bx^(Garcia-Dopico et al., 2023a); ^by^(Garcia-Dopico et al., 2023b); ^bz^(Walton et al., 2023); ^ca^(Brun et al., 2021); ^cb^(Batalla al., 2024); ^cc^(Akl et al., 2023); ^cd^(Halicka et al., 2024); ^ce^(Koumantakis et al., 2024); ^cf^(Monticone et al., 2024); ^cg^(Świdrak et al., 2023); ^ch^(Świdrak et al., 2024); ^ci^(Hardy et al., 2024);
